# p21 promotes oncolytic adenoviral activity in ovarian cancer and is a potential biomarker

**DOI:** 10.1186/1476-4598-9-175

**Published:** 2010-07-03

**Authors:** Magdalena B Flak, Claire M Connell, Claude Chelala, Kyra Archibald, Michael A Salako, Katrina J Pirlo, Michelle Lockley, Sally P Wheatley, Frances R Balkwill, Iain A McNeish

**Affiliations:** 1 Centre for Molecular Oncology and Imaging, Institute of Cancer, Barts and the London School of Medicine, Queen Mary University of London, London, EC1 M 6BQ, UK; 2 Cancer and Inflammation, Institute of Cancer, Barts and the London School of Medicine, Queen Mary University of London, London, EC1 M 6BQ, UK; 3 Genome Damage and Stability Centre, University of Sussex, Brighton, BN1 9RQ, UK

## Abstract

The oncolytic adenovirus *dl*922-947 replicates selectively within and lyses cells with a dysregulated Rb pathway, a finding seen in > 90% human cancers. *dl*922-947 is more potent than wild type adenovirus and the E1B-deletion mutant *dl*1520 (Onyx-015). We wished to determine which host cell factors influence cytotoxicity. SV40 large T-transformed MRC5-VA cells are 3-logs more sensitive to *dl*922-947 than isogenic parental MRC5 cells, confirming that an abnormal G1/S checkpoint increases viral efficacy. The sensitivity of ovarian cancer cells to *dl*922-947 varied widely: IC_50 _values ranged from 51 (SKOV3ip1) to 0.03 pfu/cell (TOV21G). Cells sensitive to *dl*922-947 had higher S phase populations and supported earlier E1A expression. Cytotoxicity correlated poorly with both infectivity and replication, but well with expression of p21 by microarray and western blot analyses. Matched p21+/+ and -/- Hct116 cells confirmed that p21 influences *dl*922-947 activity *in vitro *and *in vivo*. siRNA-mediated p21 knockdown in sensitive TOV21G cells decreases E1A expression and viral cytotoxicity, whilst expression of p21 in resistant A2780CP cells increases virus activity *in vitro *and in intraperitoneal xenografts. These results highlight that host cell factors beyond simple infectivity can influence the efficacy of oncolytic adenoviruses. p21 expression may be an important biomarker of response in clinical trials.

## Background

Oncolytic viruses multiply selectively within infected cancer cells and cause death, with release of mature viruses that infect neighbouring cells. Upon infection, the first adenoviral protein to be expressed is E1A, which is required for the efficient transcription of other viral early genes [1]. Another function is to drive infected cells into S phase by disrupting the interaction between pRb and E2F [2], allowing transactivation of genes necessary for viral DNA replication. Two E1A conserved regions are responsible for this disruption: CR2 binds with high affinity to the B-domain of the pRb pocket whilst CR1 displaces E2F from the E1A CR2/pRb complex by low affinity binding with pRb directly at the E2F binding site [3].

We have shown that the E1A CR2 deleted adenovirus *dl*922-947 has considerable activity in ovarian cancer and induces cell death through a non-apoptotic mechanism [4]. It is more potent than E1A wild-type adenoviruses and the E1B-55K mutant *dl*1520 (Onyx-015, H101) [5,6]. *dl*922-947 replicates selectively in cells with abnormalities of the Rb pathway and consequent G1-S checkpoint, findings seen in over 90% of human cancers [7]. We also showed that *dl*922-947 activity is associated with deregulation of multiple cell cycle checkpoints and that accelerated cell cycle progression enhances efficacy [8]. In ovarian cancer, multiple G1-S cell cycle abnormalities are observed [9,10]. However, it is unclear which of these are most important for determining sensitivity to *dl*922-947, nor is there a simple biomarker assay of virus activity. Clinical trials of E1A CR2-deleted adenoviruses are underway (http://www.clinicaltrials.gov reference NCT00805376), so understanding these factors will aid identification of patients most likely to respond.

Our data indicate that infectivity is not the only determinant of cell sensitivity, so we have focussed on post-infection events. There is poor correlation between extent of viral replication and cell death when comparing different cell lines. Basal expression of p21 appears an important factor in identifying cells sensitive to adenovirus cytotoxicity and correlates with expression of E1A, death *in vitro *of malignant and transformed cells and also with anti-tumour activity *in vivo*. We also show that p21 is predominantly cytosolic and is targeted for proteasomal destruction after infection. Knockdown of p21 in high-expressing cells reduces E1A expression and adenovirus activity, whilst re-expression in p21^low ^cells increases E1A expression and the cytotoxicity of both *dl*922-947 and wild-type adenovirus. Finally, we show that p21 stabilises cyclin D expression and thus promotes a cellular environment conducive to adenovirus replication.

## Results

### Oncolytic adenoviral activity correlates with E1A expression and S phase fraction but not infectivity

**Figure 1:**
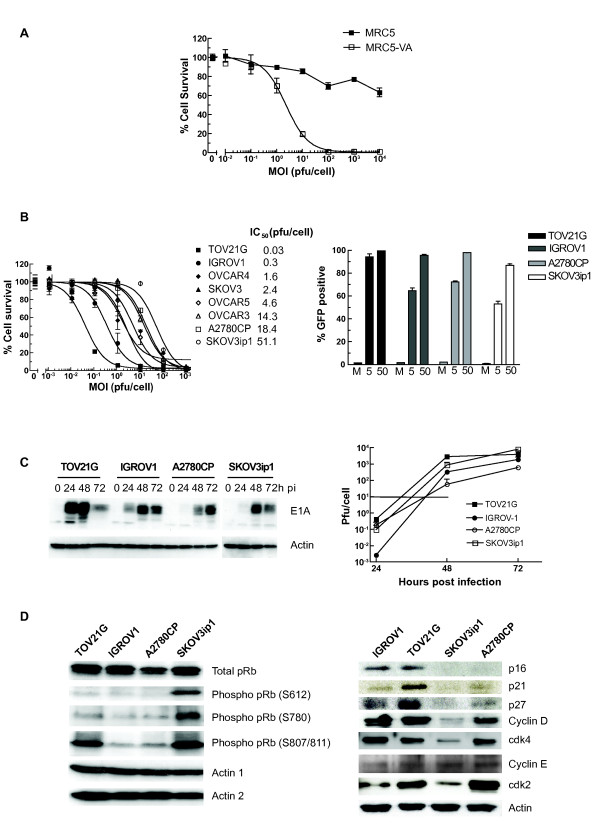
**Activity of *dl*922-947 in immortalized and ovarian cancer cell lines**. **1A**: **Cytotoxicity in MRC5 fibroblasts**. MRC5 and MRC-VA cells were infected with *dl*922-947 (MOI 0.01 - 10000 pfu/cell). Cell survival was assessed 144 hours later. **1B**: **Cytotoxicity and infectivity in ovarian cancer cells**. Eight ovarian cancer cell lines were infected with *dl*922-947 (MOI 0.001 - 1000 pfu/cell). Cell survival was assessed 120 hours later (left). Cell infectivity in TOV21G, IGROV1, A2780CP and SKOV3ip1 cells was assessed 24 h following infection with Ad-CMV-GFP (MOI 5 and 50 pfu/cell) using flow cytometry. Results are presented as percentage cells GFP positive (right). **1C: Viral protein expression and replication**. Ovarian cancer cells were infected with *dl*922-947 (MOI 10) and protein harvested up to 72 hours later. E1A expression was analyzed by immunoblot (left). TOV21G, IGROV1, A2780CP and SKOV3ip1 were infected with *dl*922-947 (MOI 10). Viral replication was assessed up to 72 hours later by and TCID_50 _assay. Horizontal lines represents input dose (right). **1D: Expression of Rb pathway components in ovarian cancer cells**. Protein was harvested from asynchronous growing ovarian cancer cells, separated on SDS-PAGE cells and analyzed by immunoblot.

We wished to determine which host cell factors contributed to cell sensitivity to the E1A CR2-deleted adenovirus *dl*922-947. We first examined normal (MRC5) and SV40 Large T-transformed (MRC5-VA) human lung fibroblasts. MRC-VA cells were dramatically more sensitive to *dl*922-947 (IC_50 _> 10^4 ^pfu/cell (MRC) vs 2.3 pfu/cell (MRC-VA)) (Fig 1A) and supported significantly greater viral replication (Additional File 1). Thus, complete deregulation of Rb pathway induced by SV40 Large T antigen has profound effects upon virus activity.

The cytotoxicity of *dl*922-947 was then assessed in a panel of eight human ovarian cancer cells (Fig 1B). The panel's sensitivity to *dl*922-947 varied greatly, with IC_50 _values ranging from 0.03 pfu/cell (TOV21G) to 51.1 pfu/cell (SKOV3ip1). We chose the two most sensitive (TOV21G and IGROV1) and least sensitive (SKOV3ip1 and A2780CP) lines for further evaluation. Since the adenovirus life cycle relies upon host cell infection, we analysed infectivity in the 4 cell-lines using two assays: GFP fluorescence following infection with non-replicating Ad CMV-GFP (Fig 1B) and qPCR for internalised viral genomes two hours after infection with *dl*922-947 (Additional File 2). Infectivity could not explain differences in virus efficacy, as there was poor correlation with IC_50 _and all 4 lines were infectable. E1A, first adenovirus gene to be expressed, is an absolute pre-requisite for productive infection. There was a clear correlation between early expression of E1A in the 4 lines and their sensitivity to viral cytotoxicity (Fig 1C) but no complete correlation between cytotoxicity and infectious virion production (Fig 1C) in this comparison of four cell lines: virion production was initially highest in TOV21G, but was exceeded in SKOV3ip1 cells at 72 hours. Meanwhile, IGROV1 cells generated fewer virions than resistant SKOV3ip1 cells at all time points. Thus, cytotoxicity appears not to be a direct function of intracellular virion number.

We chose to investigate host cell factors governing cell cycle progression and their potential role in determining viral efficacy. Cell cycle analysis of uninfected, asynchronous cells by propidium iodide flow cytometry demonstrated a strong correlation between proportion of cells in S phase and sensitivity to *dl*922-947 (r^2 ^= 0.91; p = 0.04 - Additional File 3), although S phase fraction did not translate into cell growth rate, with A2780CP being the most rapidly growing line (Additional File 3). We examined gene expression data of the five members of our ovarian cancer cells (IGROV1, OVCAR3, OVCAR4, OVCAR5, SKOV3) that are part of the NCI60 panel. Using Gene Expression Omnibus data (GEO http://www.ncbi.nlm.nih.gov/projects/geo/index.cgi), we identified genes most differentially expressed in the most sensitive of the five lines (IGROV1) compared with the others. The two most over-expressed cell cycle genes were CDKN1A (p21^Waf1^) and CCND2 (Cyclin D2 - Additional File 4). As data from MRC fibroblasts (Fig 1A) and our previous results from immortalized ovarian surface epithelial cells [6] indicated that Rb pathway status is a strong determinant of cell sensitivity to *dl*922-947, we examined expression of components of this pathway in the two sensitive and two resistant lines (Fig 1D). We observed no obvious correlation between cell sensitivity and phosphorylation of pRb at three sites (Ser 612, S780 and Ser 807/811). However, we did observe that expression of p21, as well as Cyclin D, p16 and p27, correlated well with cell sensitivity. Therefore, we investigated the role of p21 in adenoviral activity and whether its expression might act as a biomarker for *dl*922-947 activity.

### p21 expression in matched Hct116 cells

**Figure 2:**
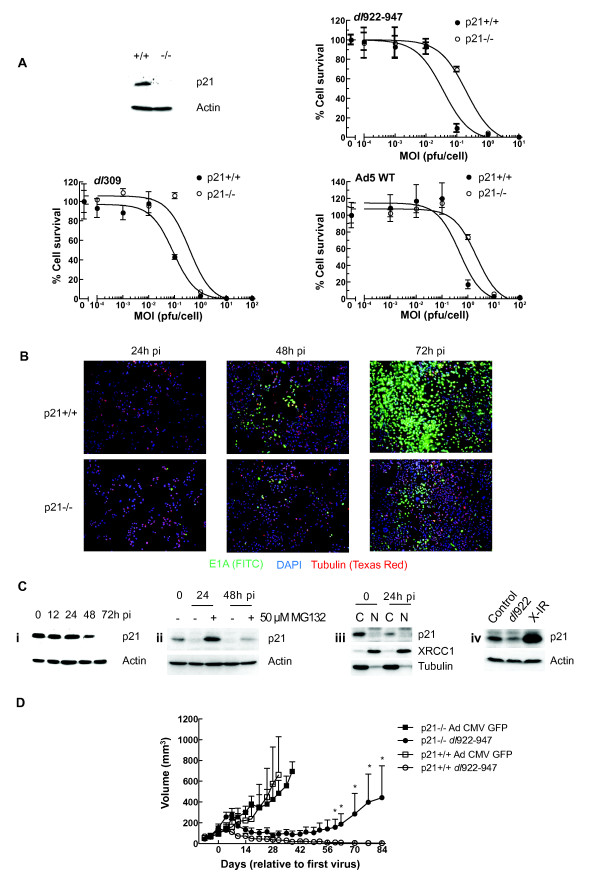
**p21 expression increases adenovirus activity**. **2A: p21 expression increases adenoviral cytotoxicity**. Hct116 p21^+/+ ^and p21^-/- ^cells were infected with *dl*922-947 (upper right), *dl*309 (lower left) and Ad5 WT (lower right) (MOI 0.0001 - 100 pfu/cell). Cell survival was assessed 144 hours later. Absence of p21 expression in uninfected p21^-/- ^cells was confirmed by immunoblot (top left). **2B: p21 increases E1A expression**. Hct116 p21^+/+ ^and p21^-/- ^cells were grown on poly-L-lysine-coated coverslips, infected with *dl*922-947 (MOI 0.5) and fixed up to 72 h pi with 5% formaldehyde. Following permeabilisation, E1A and tubulin expression was assessed by immunofluorescence. **2C: p21 undergoes proteasomal degradation following adenovirus infection**. Protein was harvested from Hct116 p21^+/+ ^cells up to 72 h post-infection with *dl*922-947 (MOI 10) and analyzed by immunoblot for p21 expression (Ci). Cells were treated with 50 μM MG132 for 6 hours prior to harvest (Cii). Hct116 p21^+/+ ^cells were also subjected to cell fractionation 24 h post-infection. C = cytoplasmic fraction, N = nuclear fraction (Ciii). p21^+/+ ^cells were also harvested 6 h following exposure to 5Gy X-irradiation (X-IR) and blotted for p21 expression (Civ). **2D: p21 expression increases *dl*922-947 activity *in vivo***. 5 × 10^6 ^Hct116 p21^+/+ ^and p21^-/- ^cells were injected subcutaneously in the flanks of CD1 nu/nu female mice. Once tumours reached approximately 100 mm^3^, *dl*922-947 or Ad CMV GFP was injected intratumorally into (1 × 10^10 ^particles in 50 μl PBS; n = 4-5 per group) on three separate occasions. Tumours were measured using callipers. Points represent mean ± s.e.m. *; p < 0.05 by unpaired, one-tailed Student's *t *test.

We first examined matched p21^+/+ ^and p21^-/- ^Hct116 cells. There was no difference in infectivity between the two cell lines (data not shown), but expression of p21 in Hct116 cells is associated with significantly increased sensitivity to *dl*922-947 and also two E1-wild-type viruses, Ad5 WT and *dl*309 (Fig 2A) in 120-hour cytotoxicity assays, although there was no difference at 72 hours pi (data not shown). In addition, p21^+/+ ^cells express more E1A (as detected by immunofluorescence - Fig 2B) and generate and release significantly more infectious virions than p21^-/- ^cells (Additional File 5). Following infection with *dl*922-947, p21 expression declined significantly between 24 and 48 hours post infection (pi) (Fig 2C), which was reversed following treatment with MG132, indicating that p21 is targeted for proteasomal destruction (Fig 2C). Sub-cellular fractionation indicated that p21 was largely cytoplasmic and unlikely, therefore, to function as a cyclin-dependent kinase inhibitor (Fig 2C). To examine how Hct116 p21^+/+ ^cells responded to other forms of genotoxic stress, we exposed them to x-irradiation. Six hours following irradiation, there is a marked increase in p21 expression, compared to the reduction seen with *dl*922-947 (Fig 2C). Finally, we implanted Hct116 p21^+/+ ^and p21^-/- ^cells in the flank of female nude mice. Once tumours were approximately 100 mm^3^, *dl*922-947 or Ad CMV-GFP (1 × 10^10 ^particles in 50 μl PBS) was injected intra-tumourally on three occasions. Both p21^+/+ ^and p21^-/- ^tumours injected with control virus continued to grow. However, following *dl*922-947 injection, p21^+/+ ^tumours were smaller than p21^-/-^, which persisted until the end of the experiment and reached statistical significance (Fig 2D).

### p21 expression in transformed ovarian surface epithelial cells

**Figure 3:**
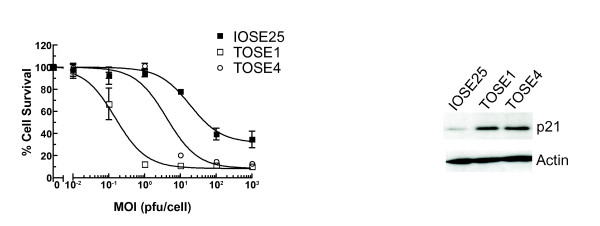
**Activity of *dl*922-947 in non-malignant human ovarian surface epithelial cells**. IOSE25, TOSE1 and TOSE4 cells were infected with *dl*922-947 (MOI 0.01 - 10000 pfu/cell). Cell survival was assessed 120 hours later (left). Expression of p21 was analyzed by immunoblot (right).

We next examined the hTERT-immortalised ovarian surface epithelial cell line IOSE25, which has intact Rb and p53 function [11]. We have recently identified two spontaneously transformed IOSE25 sub-lines, TOSE1 and TOSE4, which have acquired the ability to grow in soft agar but do not form tumours within immunocompromised mice and thus are a valuable tool for investigating early ovarian carcinogenesis (Archibald et al 2010 MS in preparation). TOSE1 and 4 are greatly more sensitive to *dl*922-947 than IOSE25 (Fig 3) and express high levels of p21.

### p21 knockdown prior to infection reduces viral activity

**Figure 4:**
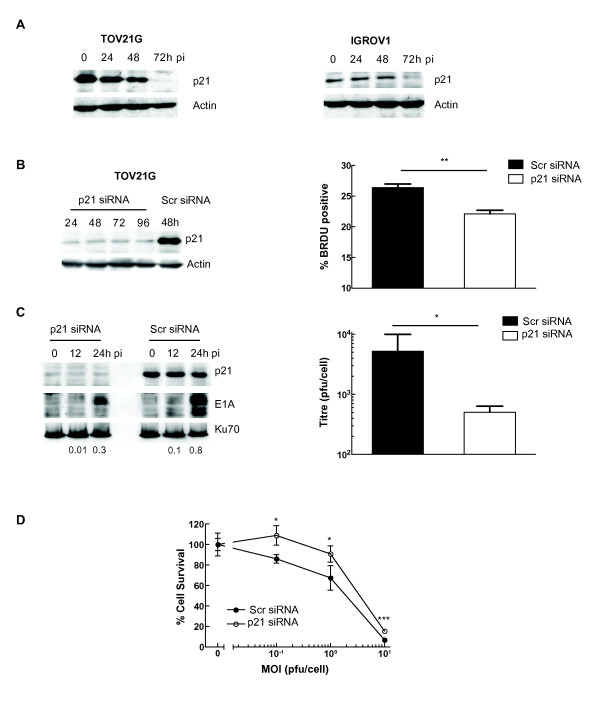
**p21 knockdown decreases *dl*922-947 activity in ovarian cancer cells**. **4A: p21 is degraded in ovarian cancer cells following adenoviral infection**. Protein was harvested from TOV21G and IGROV1 cells up to 72 h post-infection with *dl*922-947 (MOI 10) and analyzed by immunoblot for p21 expression. **4B, C and D: siRNA-mediated p21 knockdown reduces *dl*922-947 activity**. TOV21G cells were transfected with 60pmol p21 siRNA or scrambled control. Expression of p21 assessed by immunoblot up to 96 hours later (4B left). DNA replication was assessed by BRDU incorporation 36 hours following siRNA transfection. ** p = 0.001 (4B right). TOV21G cells were transfected with p21 or scrambled control siRNA and infected 24 hours later with *dl*922-947 (MOI 10). Protein was harvested up to 24 h thereafter and analyzed by immunoblot for E1A and p21 expression. Numbers below blots represent E1A:Ku70 ratio (4C left). In addition, intracellular virion production was assessed 48 h pi by TCID_50_. * p < 0.05 (4C right). TOV21G cells (10^4 ^cells/well) were transfected with 20 pmol p21 or scrambled control siRNA in 24 well plates. 24 hours later, they were infected with *dl*922-947 (MOI 0, 0.1, 1 and 10). Cell survival was assessed 96 hours later by MTT assay. * p < 0.05. *** p < 0.001.

As with the Hct116 p21^+/+ ^cells, we observed a reduction in p21 expression in the two sensitive ovarian cancer cell lines (TOV21G and IGROV1) following infection with *dl*922-947 (Fig 4A). Using a pool of four siRNA constructs, p21 knockdown was achieved in TOV21G cells for up to 96 hours, which was associated with a significant reduction in DNA replication, as detected by BRDU incorporation (TOV21G + Scr 26.4 ± 0.6% vs TOV21G + p21 siRNA 22.1 ± 0.6%, p = 0.001; Fig 4B). TOV21G cells were infected with *dl*922-947 24 hours following siRNA-mediated p21 knockdown. The p21 knockdown reduced E1A expression and infectious virion production significantly (Fig 4C). Finally, 24 hours following p21 knockdown, TOV21G cells were infected with *dl*922-947 and cytotoxicity assessed 96 hours thereafter. At all MOI studied, there was significantly less cell death in the p21 knockdown cells than in those treated with scrambled siRNA (Fig 4D).

### Re-expression of p21 augments S phase fraction and viral cytotoxicity in A2780CP cells

**Figure 5:**
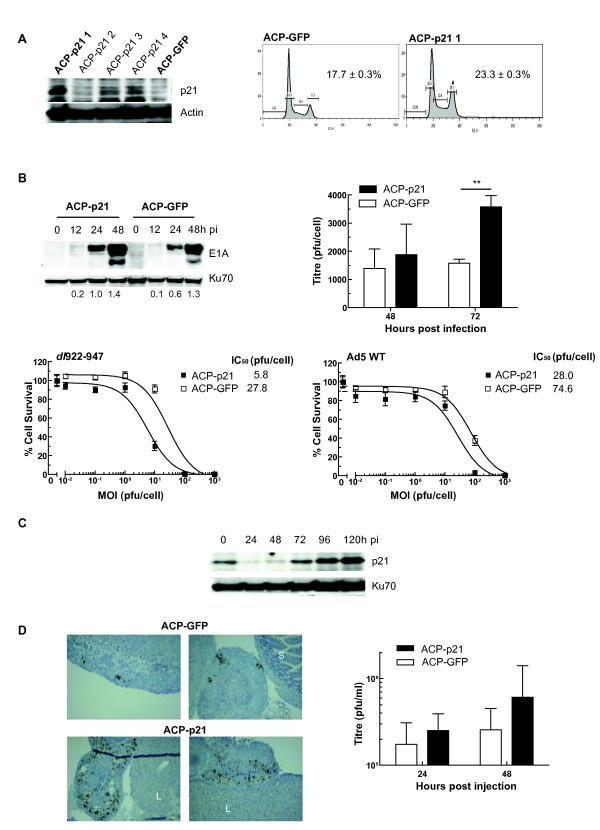
**p21 re-expression increases adenovirus activity**. **5A: Expression of p21 in A2780CP cells increases S phase fraction**. A2780CP expressing p21 cells were generated as described in Materials and Methods. Expression of p21 in one pool (ACP-p21-1) was confirmed by immunoblot (left). Cell cycle status in asynchronous ACP-p21-1 and control cells (ACP-GFP) was assessed following propidium iodide staining. Figures represent the percentage cells in S phase. **5B: p21 expression increases E1A expression, viral replication and cytotoxicity**. 10^5 ^ACP-p21 and ACP-GFP were infected with *dl*922-947 (MOI 10). E1A expression was assessed by immunoblot (top left) and intracellular virion production was assessed by TCID_50 _(top right) ** p < 0.01. ACP-p21 and ACP-GFP cells were infected with *dl*922-947 and Ad5 WT (MOI 0.01-1000). Numbers below blots represent E1A:Ku70 ratio. Cell survival was assessed 120 hours later (5B bottom left and right). **5C: p21 expression in ACP-p21 cells decreases following *dl*922-947 infection**. ACP-p21 cells were infected with *dl*922-947 (MOI 10). Expression of p21 was analyzed by immunoblot up to 120 h pi. **5D: p21 expression increases viral activity *in vivo***. Female Balb C nu/nu mice were inoculated ip with 5 × 10^6 ^ACP-p21 or ACP-GFP cells (n = 5 per group). One week later, *dl*922-947 was injected ip (5 × 10^9 ^particles daily x3). Blood was taken 24 hours after last virus injection and mice were killed 24 h thereafter. Expression of E1A was assessed by immunohistochemistry (left). All images are x100, S = Small intestine; L = Liver. Virion levels in serum were assessed by TCID_50 _(right). Results represent mean ± sd, n = 5.

We next investigated the effects of re-expressing p21 in a cell line with intrinsically low levels of the protein and low sensitivity to *dl*922-947. A2780CP cells were transfected with a plasmid encoding p21 under CMV immediate early promoter control. We selected one pool, ACP-p21 1 (Fig 5A), with stable p21 expression. As control, we used A2780CP stably expressing GFP (ACP-GFP), which expressed very low levels of p21, comparable with the parental cell line.

There was a significant increase in S phase population in asynchronous log-growth phase ACP-p21 cells compared to ACP-GFP cells (23.3 ± 0.3% vs 17.7 ± 0.3% respectively, p < 0.001) (Fig 5A). There was also a corresponding and significant increase in BRDU uptake in ACP-p21 cells compared to parental A2780CP cells (Additional File 6). Interestingly, expression of p21 appeared to induce a small, but statistically significant, inhibitory effect on infectivity (Additional File 7). However, despite this, ACP-p21 cells supported greater E1A expression 24 h pi and increased production of infectious *dl*922-947 virions and were also more sensitive to both *dl*922-947 and Ad5 WT cytotoxicity (Fig 5B). As with Hct116 p21^+/+^, TOV21G and IGROV1 cells, p21 expression diminished 24-48 hours pi in ACP-p21 cells, but rose again 72 and 120 h pi (Fig 5C).

We then implanted ACP-p21 and ACP-GFP cells intraperitoneally (i.p.) in female nude mice. These xenografts are extremely aggressive, with untreated animals requiring sacrifice after 18-20 days (data not shown). Mice were inoculated i.p. with *dl*922-947 on days 8-10 inclusive and sacrificed 48 hours thereafter. p21-expressing xenografts supported greater E1A expression within tumour nodules (Fig 5D). There were also consequent increases in infectious virion titre in serum in ACP-p21 bearing mice compared to ACP-GFP mice, although the differences did not reach statistical significance - ACP-GFP mean titres 24 and 48 hours pi: 1.74 × 10^5 ^+/- 1.36 × 10^5 ^and 2.56 × 10^5 ^+/- 1.95 × 10^5 ^pfu/ml (mean +/- s.d. n = 5) respectively. For ACP-p21 cells, the titres at the same time points were 2.52 × 10^5 ^+/- 1.42 × 10^5 ^and 6.08 × 10^5 ^+/- 6.95 × 10^5 ^pfu/ml.

### p21 stabilizes cyclin D

**Figure 6:**
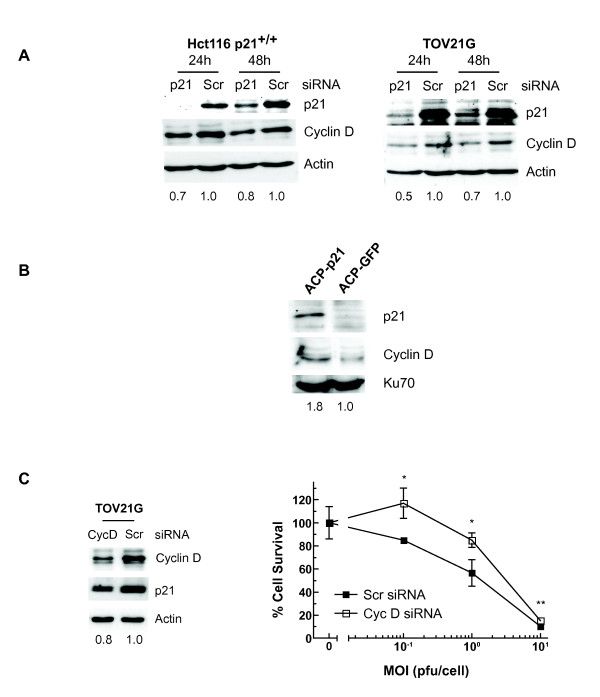
**p21 stabilizes cyclin D prior to infection**. **6A: p21 knockdown reduces cyclin D expression**. Protein was harvested from Hct116 p21^+/+ ^and TOV21G cells up to 48 h followed treatment with 60 pmol p21 or scrambled control siRNA. Expression of p21 and cyclin D was assessed by immunoblot. Numbers represent Cyclin D:actin ratio, normalised to Scr-treated controls. **6B: p21 re-expression increases cyclin D**. Protein was harvested from 10^5 ^ACP-p21 and ACP-GFP cells. Cyclin D and p21 expression was assessed by immunoblot. Numbers represent Cyclin D:Ku70 ratio, normalised to ACP-GFP controls. **6C: Cyclin D knockdown reduces p21 expression and reduces cytotoxicity**. Protein was harvested from TOV21G cells 24 h followed treatment with 60 pmol cyclin D or scrambled control siRNA. Expression of p21 and cyclin D was assessed by immunoblot. Numbers represent p21:actin ratio, normalised to Scr-treated controls. TOV21G cells (10^4 ^cells/well) were also transfected with 20 pmol cyclin D or scrambled control siRNA in 24 well plates. 24 hours later, they were infected with *dl*922-947 (MOI 0, 0.1, 1 and 10). Cell survival was assessed 96 hours later by MTT assay. * p < 0.05. ** p < 0.01.

Finally, we investigated the mechanism by which p21 expression could be associated with increased adenoviral activity. We first examined endogenous activity of the PI3kinase/AKT pathway in the four ovarian cancer cell lines. Phosphorylation of p21 at Thr145 by AKT reduces the binding of p21 to cdk2 and promotes cell cycle progression [12]. Thus, it remained possible that any p21 expressed in TOV21G and IGROV1 cells was unable to exert cell cycle inhibitory effects. Levels of activated AKT (Ser 473 phospho-AKT) in uninfected cells were highest in TOV21G cells, but were largely similar in the other three lines (Additional File 8), whilst knockdown of p21 in TOV21G cells had no effect on upstream PI3kinase activation (data not shown). However, we did observe that basal cdk2 kinase activity was greatest in p21-expressing TOV21G and IGROV1 cells, as determined by phosphorylation of Histone H1 in asynchronous cells (Additional File 9), in keeping with the greater rates of S phase seen as detected by flow cytometry. In addition, there was a reduction in cyclin D expression in uninfected Hct116 p21^+/+ ^and TOV21G cells following p21 knockdown (Fig 6A), suggesting that cyclin D stability is at least partially p21-dependent in these cells. Conversely, we also observed an increase in cyclin D expression in ACP-p21 cells compared to ACP-GFP (Fig 6B), and, when cyclin D was knocked down in TOV21G cells by pools of siRNA directed against all three isoforms, there was a parallel reduction in p21 expression and a significant reduction in *dl*922-947 cytotoxicity (Fig 6C). Thus, p21 and cyclin D appear to be stabilise each other and co-operate to promote virus activity.

## Discussion

A key step in development of novel cancer therapies is the identification of biomarkers that can predict which patients might respond to treatment. Our results suggest that expression of p21 may be a predictive biomarker for the oncolytic adenovirus *dl*922-947. Many investigators have focused on expression of CAR (Coxsackie Adenovirus Receptor), the primary receptor for Group C adenovirus, as the most important determinant of adenovirus function [13]. Undoubtedly, the ability of virus to infect the host cell is vital for subsequent activity. However, the largest study of primary ovarian cancers reveals that the majority retain expression of CAR [14], whilst primary ascitic cells can show demonstrable CAR and α_v_β_3/5 _integrin expression [15]. We show that infectivity alone is a poor predictor of cell sensitivity to virus-induced death. It is also clear that the process of adenovirus infection involves receptors and co-receptors other than classical CAR and α_v_β_3/5 _integrins, [16,17].

The first adenoviral gene to be expressed is E1A, which relies upon host cell transcription factors, including EF-1A, E2F and Sp1 [18,19]. As E1A expression correlates directly with overall virus efficacy, the ability of the host cell to permit expression will profoundly influence all subsequent parts of the lifecycle. We show that p21 expression in uninfected cancer cells is associated with such a permissive state in cancer cells. One function of E1A is to induce infected cells into S phase, the phase most conducive to viral DNA replication. The two cells lines that were most sensitive to viral efficacy expressed p21 prior to infection and had the highest rate of S phase in uninfected asynchronous populations, although this did not correlate with rate of growth *in vitro *or *in vivo *or the state of pRb phosphorylation. pRb is phosphorylated on multiple sites by cyclin/cdk complexes [20], with individual complexes preferentially phosphorylating different residues to alter function [21-23]. Hyperphosphorylation of pRb late in G1 heralds transcription of genes necessary for host cell (and viral) DNA replication. However, there is now evidence that pRb can be phosphorylated by kinases other than cdks [24] and that p21 itself can bind to pRb and alter phosphorylation [25], rendering patterns of pRb phosphorylation in asynchronous cells a poor predictor of virus activity. In addition, p21 binds to the same A/B pocket in pRb as E2F and may thus displace E2F independently of pRb phosphorylation [25].

p21 has a multiple roles within cells. Whilst it is a cdk inhibitor (CKI) and a single molecule can completely inhibit cyclin A/cdk1 activity [26], it functions as far more than a pure CKI; firstly, members of the p21 family act as assembly factors, promoting the formation of active cyclin D/cdk4 complexes at low concentrations, also stabilising cyclin D [27,28]. Our results show that p21 knockdown causes loss of cyclin D expression in uninfected TOV21G and Hct116 cells, whilst its re-expression in ACP-p21 cells increases cyclin D levels. Conversely, knockdown of cyclin D causes a reduction in p21 levels, confirming the interdependency of the two in the absence of genotoxic stress. Secondly, binding of p21 to cyclin D/cdk4 complexes titrates p21 away from cyclin E/cdk2, thus promoting S phase entry [29]. Our data show that p21 is predominantly cytoplasmic, and thus is titrated away from cyclin E/cdk2 to facilitate viral activity. In addition, the two sensitive ovarian cell lines demonstrated greater phosphorylation of histone H1 in basal conditions. This histone is phosphorylated by cdk2 at G1/S transition [30] and is thus a marker of cdk2 kinase activity. Previous studies have indicated that Akt-mediated p21 phosphorylation can result in p21 localizing to the cytoplasm in Her2-positive cancer cells [31] and also inhibit p21 binding to cdk2 [12]. Our results indicate that the most sensitive line, TOV21G, had higher levels of basal AKT (S473) phosphorylation than the other lines, but there was no overall correlation between cell sensitivity and PI3kinase/AKT activity. Finally, p21, when phosphorylated at Thr57 by cdk2, has a role in promoting association between cdc2 (cdk1) and cyclin B and hence facilitates G2/M progression [32]. We have previously shown that *dl*922-947 is capable of over-riding multiple cell cycle checkpoints in sensitive cells. This ability is augmented by nuclear expression of survivin, which acts to augment cyclin D activity [8].

In response to genotoxic stress, p21 translocates to the nucleus where it causes cell cycle arrest and promotes nucleotide excision repair following association with PCNA [33]. We show that p21 expression falls following adenovirus infection, with evidence of proteasomal degradation. Expression of E1A alone increases p21 expression through direct transactivation of the p21 promoter [34]. However, any increase in p21 significantly above basal levels would promote its ability to arrest the cell cycle, to the detriment of adenoviral function. Thus, p21 levels fall post-infection. The adenovirus proteins E1B-55K and E4orf6 form the core of an E3 ubiquitin ligase complex that targets cellular proteins, including p53, Mre11 and DNA ligase IV, for proteasomal destruction [35]: p21 may also be a target for this complex. Interestingly, expression of p21 fell only transiently in the ACP-p21 cells, where expression is under the control of a constitutive promoter, suggesting that some p21 loss in Hct116 and TOV21G cells may result from reduced transcription following the destruction of p53 by E1B55K/E4orf6.

Two recent publications have suggested that p21 expression might reduce oncolytic adenovirus activity in cancer cells, including Hct116. Like us, Höti et al [36] found that there was greater expression of E1A in Hct116 p21^+/+ ^cells than in p21^-/-^. However, they found that treatment of cells with valproic acid, a pan-HDAC inhibitor, reduced oncolytic adenovirus activity and was associated with an increase in p21 expression. Recent evidence suggests that the number of genes responsive to valproic acid is at least 100 and may exceed 1000 [37,38], whilst the pathways inhibited by valproic acid in myeloma cells include not only cell cycle progression, but also DNA replication and gene transcription [39], all of which are required for adenovirus function. In addition, valproic acid will inhibit HDAC3, which has a critical role in S phase progression [40]. Together, these findings suggest strongly that the effects of valproic acid are not mediated purely by p21. Finally, Höti et al indicated that co-infection of Hct116 p21^-/- ^cells with an oncolytic virus and a non-replicating virus expressing p21 reduced oncolytic virus replication compared to co-infection with a control virus. However, such treatment will force an increase in p21 expression after infection (and thus block cell cycle progression), which is the opposite of the natural expression pattern - like us, Höti et al and others [41] have shown that p21 expression falls after infection; we wish to show in our experiments that it is expression of p21 *prior *to infection that is relevant. In the second manuscript [42], Hct116 p21^+/+ ^and p21^-/- ^cells were infected with a variety of oncolytic adenoviruses, including another E1A CR2-deleted virus Δ24. Results suggested that there was greater anti-tumour efficacy in p21^-/- ^cells or in cells in which p21 was knocked down via siRNA. However, dose response experiments were performed only at a single early time point (72 hours) after infection and the lowest MOI employed is 1 pfu/cell: in our experiments, the difference in efficacy of both *dl*922-947 and the wild-type viruses became evident 120-144 hours post-infection and the IC_50 _values of 0.01-0.1 were seen; thus Shiina et al will have missed significant differences at lower doses and/or later time points. Also, following siRNA-mediated p21 knockdown, cells were re-seeded prior to infection with adenoviruses, which could significantly alter expression of a cell cycle-related gene even after RNAi; survival is then assessed after exposure to only a single dose of virus, rather than a formal dose response range. There is no assessment of virus protein expression, change in p21 expression following infection or *in vivo *assessment of the role of p21, nor any demonstration of the effect of p21 re-expression in cells with low endogenous expression. Finally, Δ24 was generated using the adenovirus plasmid pBHG10 [43], which lacks the entire E3 region, including E3-11.6 Adenovirus Death Protein (ADP), whilst *dl*922-947 has intact ADP. Although the mechanism by which ADP promotes cell death late after infection is unclear, we and others have shown that deletions or mutations within ADP certainly alter the kinetics of adenovirus-induced death [4,44] and also impair virus spread [45]. Thus we believe that our thorough examination both *in vitro *and *in vivo *does support a role for basal p21 expression in cancer cells prior to infection in promoting an environment conducive to viral replication.

There are conflicting data on p21 in ovarian cancer. In the serous sub-type, p21 expression is frequently lost [46] and this appears to be a poor prognostic factor [47]. However, in clear cell carcinoma, which has low response rates to chemotherapy and poor overall prognosis [48], p21 expression is frequently seen [46,49]. It is noteworthy that TOV21G, our most sensitive line and which expresses high levels of p21, was derived from a clear cell tumour [50]. In addition, low malignant potential (borderline) ovarian tumours are characterized by p21 expression [51], and our results with TOSE cells, which are transformed but cannot form tumours in nude mice, are consistent with this finding.

Although there are likely to be many potential biomarkers of adenoviral activity, these results indicate that basal expression of p21 in ovarian cancer prior to infection is associated with an environment conducive to oncolytic adenoviral activity and might have use as a biomarker in future clinical trials.

## Methods

### Cell Culture and Cell Viability Assays

All cancer cells were maintained at 37°C with 5% CO_2_, in Dulbecco's modified Eagle's medium, supplemented with 10% foetal calf serum (FCS), penicillin/streptomycin and fungizome. Details of cell line origin and authentication are found in Additional File 10. IOSE25, TOSE1 and TOSE4 cells were maintained in NOSE-CM medium, as previously described [52]. A2780CP-p21 (ACP-p21) cells were generated following transfection of A2780CP cells with pCEP-WAF1 (AdGene, Cambridge, MA) using FuGene6 (Roche) followed by selection in 200 μg/ml hygromycin. For viability assays, 2 × 10^4 ^cells were infected in serum-free medium at multiplicities of infection (MOI) 0.001-1000 plaque-forming units (pfu)/cell. After 2 hours, cells were re-fed with medium containing 5% FCS. Cell viability was assayed by MTT assay using a Victor3 plate reader (Perkin Elmer, Beaconsfield, UK). All viability assays were done in triplicate and experiments repeated at least twice. For siRNA experiments, cells were transfected with ON-TARGETplus SMARTpool siRNAs or scrambled siRNA control (Dharmacon, Lafayette, CO) using DharmaFECT1. 24 hours after addition of RNAi, knockdown was confirmed by immunoblotting. Virus infection took place 24 hours after knockdown. For cyclin D siRNA, cells were transfected with equal quantities of ON-TARGETplus SMARTpool siRNAs directed against CCND1, 2 and 3. Cells were exposed to 5 Gy X-irradiation using an Hs-X-Ray System (A.G.O. Installations Ltd., Reading, UK).

### Cellular Fractionation

Cells were washed in PBS and re-suspended in ice-cold buffer I (0.3 M sucrose, 150 mM NaCl, 5 mM MgCl_2_, 0.1 mM EGTA, 15 mM Tris.HCl pH7.5, 0.5 mM DTT, plus protease inhibitors). An equal volume of buffer II (buffer I plus 4% IGEPAL) was added and the mixture incubated on ice for 10 mins before layering onto sucrose (buffer I containing 1.2 M sucrose). Samples were centrifuged at 10,000 × *g *for 20 min at 4°C. Supernatant (cytoplasmic fraction) was harvested, and nuclei lysed in RIPA buffer (20 mM Tris (pH 8.0), 137 mM NaCl, 0.5 mM EDTA, 10% glycerol, 1% nonidet-P40, 0.1% SDS, 1% deoxycholate, plus protease inhibitors, Benzonase and 2 mM MgCl_2_).

### Immunoblotting and immunofluorescence

Protein lysates were electrophoresed on SDS-polyacrylamide gels and transferred onto nitrocellulose membranes by semi-dry blotting. Antibody binding was visualized using enhanced chemiluminescence (GE Healthcare, Buckinghamshire, UK). Antibodies used were anti-E1A (Santa Cruz Biotechnology), anti-p21, anti-p27, anti-cyclin E, anti-cyclin D, anti-cdk4 (all BD Biosciences), anti-phosphorylated pRb (Ser807/811 New England Biolabs; Ser780 and Ser612 Abcam), anti-adenovirus (Abcam), anti-Ku-70 and anti-actin (Santa Cruz Biotechnology). For immunofluorescence, Hct116 cells were grown on poly-L-lysine-coated coverslips, infected with *dl*922-947 (MOI 0.5) and fixed with 5% formaldehyde. Cells were permeabilised with 0.15% Triton X-100 and primary antibody binding visualized with Texas red or Fluorescein-conjugated secondary antibodies (Vector Laboratories). Coverslips were mounted in 4',6-diamidino-2-phenylindole (DAPI)-containing Vectashield and viewed using a Zeiss Axioplan2 fluorescence microscope with a 10× objective lens and digital camera (Hamamatsu, Orca-ER). Data were processed using Simple PCI software.

### Flow Cytometry

For infectivity assays, cells were infected with Ad CMV-GFP (MOI 5 and 50), typsinised 24 h pi, washed twice in ice cold PBS and re-suspended in 500 μl PBS. For cell cycle analyses, cells were infected with *dl*922-947, trypsinised, washed twice in ice cold PBS and fixed in 70% ethanol. Cells were then washed with PBS and re-suspended in 200 μl typsinised propidium iodide and 100 μg/ml RNase A (MP Biomedicals, UK). For BRDU analysis, cells were incubated with 10 μM BRDU for 1 hour, harvested, washed and fixed in ice cold 70% ethanol. After incubation with primary anti-BRDU mAb (Becton Dickinson) and FITC-conjugated anti-mouse secondary for 20 minutes each at 37°C in the dark, cells were counterstained with PI. Cells were analyzed using a flow cytometer (BD FACSCalibur™, BD Biosciences) with FlowJo software 8.8.4 (Tree Star, Ashland, OR) or a Fluorescence Activated Cell Sorter (FACSCanto, BD Biosciences) with FACS Diva software.

### *In vivo *analyses and immunohistochemistry

5 × 10^6 ^Hct116 cells were inoculated subcutaneously onto the flank of female CD1 nu/nu mice on day 1. Once tumours reached approximately 100 mm^3^, *dl*922-947 was injected intra-tumorally (1 × 10^10 ^particles daily on days 1, 4 and 7). Tumour size was measured weekly using callipers and tumour volumes calculated as follows: Volume = (l^2 ^× w)/6 × Π, where l = longest length of the tumour and w = perpendicular width. For A2780CP xenografts, 5 × 10^6 ^cells were inoculated intraperitoneally (ip) into female Balb C nu/nu mice. On day 8, *dl*922-947 was injected ip (5 × 10^9 ^particles daily for 3 days in 400 μl 20% icodextrin). 100 μl blood was taken 24 hours following the last virus injection and mice were killed 24 hours thereafter. Tumour and livers were harvested and fixed in 10% formaldehyde. 4 μm sections were cut and processed. E1A expression was detected using a rabbit anti Ad2 E1A Ab (Santa-Cruz).

### Quantitative PCR and TCID_50 _assays

Real-time PCR was performed on an ABI Prism 7700 (Applied Biosystems, Foster City, CA, USA). Oligonucleotides and probes designed for the E1A region were as follows: Sense primer: 5'-CCACCTACCCTTCACGAACTG; Anti-sense primer: Anti-sense Primer: 5'-GCCTCCTCGTTGGGATCTTC; Probe ATGATTTAGACGTGACGGCC. PCR conditions were: 50°C for 2 minutes, 95°C for 10 minutes, followed by 40 cycles of 95°C for 15 seconds and 60°C for 60 seconds. A standard curve using 10^3^-10^9 ^viral DNA genomes was used for quantification. For TCID_50 _assays, 10^5 ^cells were infected at MOI 10 pfu/cell. Cells were harvested into 0.5 ml 0.1 M Tris pH 8.0 and subjected to three rounds of freeze/thawing (liquid N_2_/37°C), after which they were centrifuged. The supernatant was titred on JH293 cells by serial dilution. To assay viral release from infected cells, culture medium was removed from cells every 24 hours and titred separated on JH293 cells.

### Microarray analysis of cells in NCI60 panel

NCI60 ovarian cancer data (GEO accession numbers: GSM35955 (IGROV1), GSM35956 (OVCAR3), GSM35957 (OVCAR4), GSM35958 (OVCAR5), GSM35960 (SKOV3)) were analyzed using Bioconductor http://www.bioconductor.org/ packages within the open source R statistical environment http://www.r-project.org. After intra-array loess normalization, Limma [53] was used for differential expression analysis. Genes differentially regulated in the most sensitive line (GSM35955 IGROV1) versus the others were identified.

### Statistical analyses and image analysis

All graphs and statistical analyses were generated using Prism4 for Mac (GraphPad, La Jolla, CA). Unless otherwise stated, all results are presented as mean+/-sd, n = 3 and all statistical analyses are unpaired, two-tailed Student's *t *test, where p < 0.05 is considered statistically significant. Immunoblot images were scanned and band density of defined regions of interest in inverted jpg images was measured using ImageJ software.

## Competing interests

The authors declare that they have no competing interests.

## Authors' contributions

MBF performed the bulk of the experiments; CMC performed experiments for figure 2 in the lab of SPW; CC performed the expression analysis from GEO database; KA generated the IOSE and TOSE cell lines in the lab of FRB; KJP performed experiments shown in figure 6 and Additional Files 6, 7, 8 and 9; MAS and ML contributed results to figure 1B; IAM conceived the project, obtained funding, performed the *in vivo *experiments and wrote the manuscript. All authors have read and approved the manuscript.

## Supplementary Material

Additional file 1 **Supplementary figure 1**. Q-PCR and TCID50 assays in MRC5 and MRC5-VA cells infected with *dl*922-947. Click here for file

Additional file 2 **Supplementary figure 2**. Infectivity of 4 ovarian cancer cells lines as assessed by TCID50 2 hours following infection with *dl*922-947. Click here for file

Additional file 3 **Supplementary figure 3**. Correlation between S phase fraction in log-growth phase ovarian cells and sensitivity to *dl*922-947. Also, ovarian cancer cell growth rate *in vitro *over 72 hours. Click here for file

Additional file 4 **Supplementary table 1**. List of top 100 differentially expressed genes in IGROV1 cells compared to other ovarian cancer cells in NCI60 panel (OVCAR3, OVCAR4, OVCAR5, SKOV3) ranked by B value. Click here for file

Additional file 5 **Supplementary figure 4**. Replication of *dl*922-947 in HCT116 p21^+/+ ^and p21^-/- ^cells. Click here for file

Additional file 6 **Supplementary figure 5**. BRCU incorporation in A2780CP and A2780P-p21 cells. Click here for file

Additional file 7 **Supplementary figure 6**. Infectivity of A2780CP and A2780CP-p21 cells. Click here for file

Additional file 8 **Supplementary figure 7**. Basal AKT phosphorylation in four ovarian cancer cell lines. Click here for file

Additional file 9 **Supplementary figure 8**. Basal Histone H1 phosphorylation in four ovarian cancer cell lines Click here for file

Additional file 10 **Supplementary materials and methods**. Method for acid histone extraction as well as techniques used for cell line verification. Click here for file
