# Supplementary material for: p21 promotes oncolytic adenoviral activity in ovarian cancer and is a potential biomarker
Source: Mol Cancer. 2010 Jul 3;9:175. doi: 10.1186/1476-4598-9-175 (PMC2904726; doi:10.1186/1476-4598-9-175)
Supplement: Additional file 1 — Supplementary figure 1. Q-PCR and TCID50 assays in MRC5 and MRC5-VA cells infected with dl922-947. [file 1476-4598-9-175-S1.PDF]

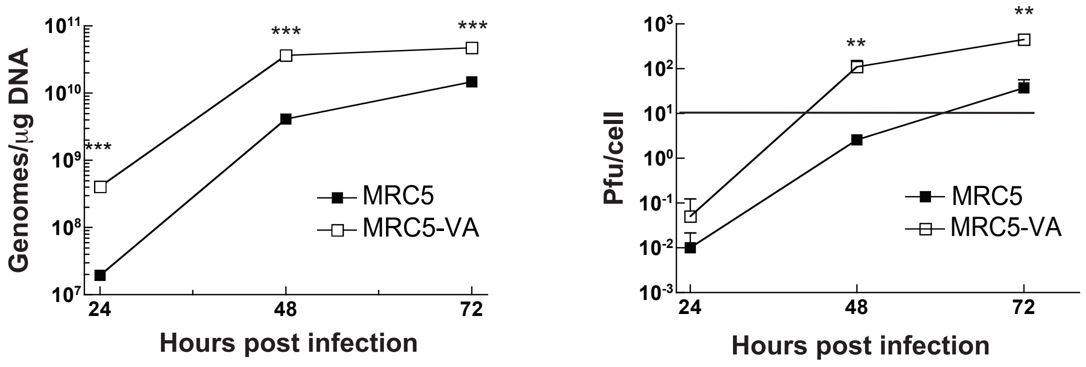

Supplementary Figure 1: MRC5 and MR5-VA cells were infected with *dl*922-947 (MOI 10).
Viral replication was assessed up to 72 hours later by Quantitative PCR (left) and TCID50 assay (right).
Horizontal line represents input dose. ** $p<0.01$. *** $p<0.001$
