# Supplementary material for: p21 promotes oncolytic adenoviral activity in ovarian cancer and is a potential biomarker
Source: Mol Cancer. 2010 Jul 3;9:175. doi: 10.1186/1476-4598-9-175 (PMC2904726; doi:10.1186/1476-4598-9-175)
Supplement: Additional file 2 — Supplementary figure 2. Infectivity of 4 ovarian cancer cells lines as assessed by TCID50 2 hours following infection with dl922-947. [file 1476-4598-9-175-S2.PDF]

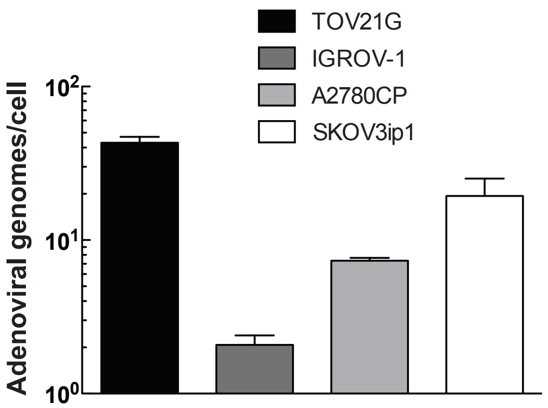

Supplementary Figure 2: TOV21G, IGROV1, A2780CP, SKOV3ip1 cells were infected with *dl*922-947 (MOI 10; approximately 100 viral particles) for two hours. Cells were washed three times then harvested into 0.5ml 0.1M Tris pH 8.0 and subjected to three rounds of freeze/thawing (liquid N2/37°C). Internalised viral genomes were quantified by Quantitative PCR.
