# Supplementary material for: p21 promotes oncolytic adenoviral activity in ovarian cancer and is a potential biomarker
Source: Mol Cancer. 2010 Jul 3;9:175. doi: 10.1186/1476-4598-9-175 (PMC2904726; doi:10.1186/1476-4598-9-175)
Supplement: Additional file 3 — Supplementary figure 3. Correlation between S phase fraction in log-growth phase ovarian cells and sensitivity to dl922-947. Also, ovarian cancer cell growth rate in vitro over 72 hours. [file 1476-4598-9-175-S3.PDF]

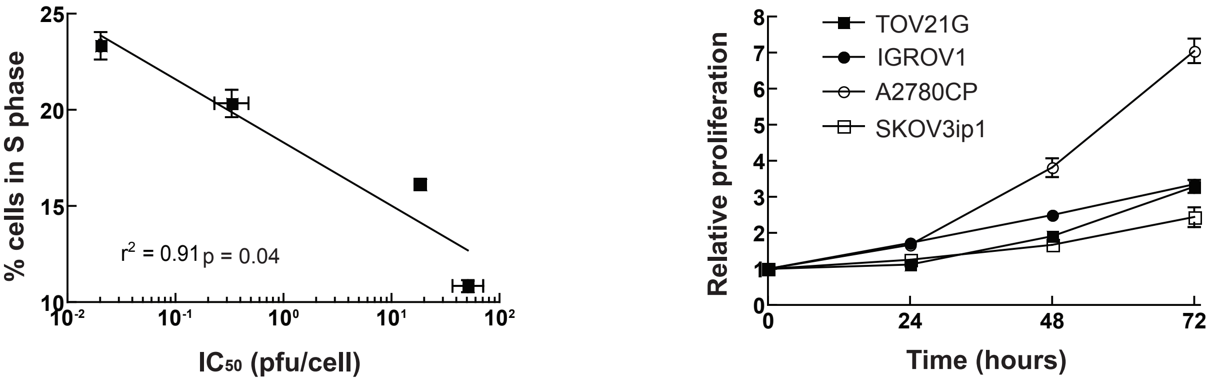

Supplementary Figure 3: Asynchronous log-growth phase TOV21G, IGROV1, A2780CP, SKOV3ip1 cells were harvested, fixed in ice-cold 70% ethanol, stained with propidium iodide and analysed for cell cycle status by flow cytometry in triplicate. Percentage cells in S phase was plotted against *dl*922-947 IC50 value (left). Cells were also plated on 24 well plates (10^4 cells/well) in triplicate and proliferation assessed by sequential MTT assays over 72h (right).
