# Supplementary material for: p21 promotes oncolytic adenoviral activity in ovarian cancer and is a potential biomarker
Source: Mol Cancer. 2010 Jul 3;9:175. doi: 10.1186/1476-4598-9-175 (PMC2904726; doi:10.1186/1476-4598-9-175)
Supplement: Additional file 4 — Supplementary table 1. List of top 100 differentially expressed genes in IGROV1 cells compared to other ovarian cancer cells in NCI60 panel (OVCAR3, OVCAR4, OVCAR5, SKOV3) ranked by B value. [file 1476-4598-9-175-S4.PDF]

Supplementary table 1
List of top 100 differentially expressed genes in IGROV1 cells compared to other ovarian cancer cells in NCI60 panel (OVCAR3, OVCAR4, OVCAR5, SKOV3) ranked by B value.

The summary table contains the following information:
M-value (M) is the log2-fold change,
A-value (A) is the average expression value accross all arrays and channels,
The moderated t-statistic (t) is the M-value to its standard error,
p-value (P) is obtained from the distribution of the moderated t-statistic (the-lower-the-better)
B-value (B) is the empirical Bayes log-odds of differential expression (the-higher-the-better).

Top two cell cycle-related genes are ranked 66 (CDKN1A; p21, Cip1) and 97 (CCND2; cyclin D2)

**Rank**	**ID_REF**	**M**	**A**	**t**	**P.Value**	**adj.P.Val**	**B**	**Platform_CLONEID**	**CLID**	**CloneID**	**UGCluster**	**Name**	**Symbol**	**Chromosome**	**Cytoband**
1	151	5.671380228	11.67813681	27.00799734	1.31E-08	9.32E-05	9.084511338	IMAGE:73185	73185	IMAGE:73185	Hs.291587	AT rich interactive domain 1B (SWI1-like)	ARID1B	6	6q25.1
2	7856	4.80439566	10.34111569	24.24928433	2.87E-08	9.32E-05	8.666013917	IMAGE:470031	470031	IMAGE:470031	Hs.466804	Phospholipase A2, group IIA (platelets, synovial fluid)	PLA2G2A	1	1p35
3	3248	5.571912544	11.03964214	24.2347401	2.89E-08	9.32E-05	8.663554581	IMAGE:245868	245868	IMAGE:245868	In multiple clusters				
4	4415	3.894634111	10.39812323	21.58366791	6.67E-08	0.000161736	8.162133255	IMAGE:293514	293514	IMAGE:293514	In multiple clusters				
5	4519	4.909677724	10.62004913	17.67883109	2.81E-07	0.000451792	7.179306851	IMAGE:296310	296310	IMAGE:296310	Hs.136102	Zinc finger CCCH-type containing 13	ZC3H13	13	13q14.12
6	2383	3.949773742	10.09416958	17.64909246	2.85E-07	0.000451792	7.170412415	IMAGE:183950	183950	IMAGE:183950	Hs.643513	Thy-1 cell surface antigen	THY1		11q22.3-q23
7	269	4.521309933	8.265406032	17.31570484	3.26E-07	0.000451792	7.068997241	IMAGE:81641	81641	IMAGE:81641	Hs.584784	Nucleoporin 88kDa	NUP88	17	17p13.2
8	7768	4.057107923	11.59184441	15.25049892	8.09E-07	0.000980458	6.363979738	IMAGE:469822	469822	IMAGE:469822	Hs.347991	Nuclear receptor subfamily 2, group F, member 2	NR2F2	15	15q26
9	7104	4.689243855	9.239771642	13.93274147	1.54E-06	0.001224598	5.833757291	IMAGE:416347	416347	IMAGE:416347	Hs.120950	Rh-associated glycoprotein	RHAG	6	6p21.1-p11
10	180	3.393542651	9.469604627	13.86714845	1.59E-06	0.001224598	5.805483399	IMAGE:74070	74070	IMAGE:74070	Hs.632456	Endosulfine alpha	ENSA		1q21.2
11	4132	4.933782271	10.66908458	13.82162145	1.63E-06	0.001224598	5.785747594	IMAGE:287028	287028	IMAGE:287028	Hs.9333	Phosphodiesterase 8A	PDE8A	15	15q25.3
12	6802	3.818453088	9.603793703	13.805081	1.64E-06	0.001224598	5.778554636	IMAGE:380053	380053	IMAGE:380053	Hs.75819	Glycoprotein M6A	GPM6A	4	4q34
13	20	5.920035972	10.91107106	17.82873833	1.20E-06	0.001224598	5.652833861	IMAGE:61196	61196	IMAGE:61196	Hs.490203	Caldesmon 1	CALD1	7	7q33
14	631	4.470121265	11.6583378	13.31954255	2.12E-06	0.001465122	5.56188116	IMAGE:125308	125308	IMAGE:125308	Hs.458272	Myeloperoxidase	MPO	17	17q23.1
15	4919	3.380357391	10.48728467	13.05157274	2.44E-06	0.001578571	5.437563747	IMAGE:308924	308924	IMAGE:308924	Hs.211831	Hemoglobin, epsilon 1	HBE1	11	11p15.5
16	8047	4.204174096	9.297965251	12.71380881	2.94E-06	0.001780497	5.27583349	IMAGE:471125	471125	IMAGE:471125	Hs.364941	Hydroxy-delta-5-steroid dehydrogenase, 3 beta- and steroid delta-isomerase 1	HSD3B1	1	1p13.1
17	4960	2.132827076	10.2297221	11.8453811	4.83E-06	0.002754686	4.832458582	IMAGE:309395	309395	IMAGE:309395	Hs.437241	ATPase, Class V, type 10D	ATP10D	4	4p12
18	176	3.796277325	10.28385777	11.09615657	7.62E-06	0.003823849	4.415058052	IMAGE:74275	74275	IMAGE:74275	In multiple clusters				
19	5058	2.485157933	9.808401087	10.96546499	8.28E-06	0.003823849	4.33865825	IMAGE:321203	321203	IMAGE:321203	Hs.504352	Neurotrimin	HNT	11	11q25
20	9201	2.466928991	8.818420577	10.94761776	8.37E-06	0.003823849	4.328138745	IMAGE:489175	489175	IMAGE:489175	Hs.558296	Acid phosphatase 1, soluble	ACP1	2	2p25
21	6558	3.21859264	11.18774487	10.91549974	8.55E-06	0.003823849	4.309155027	IMAGE:375834	375834	IMAGE:375834	Hs.102267	Lysyl oxidase	LOX	5	5q23.2
22	6813	2.307147646	12.05749317	10.89143999	8.68E-06	0.003823849	4.294889672	IMAGE:380294	380294	IMAGE:380294	Hs.496512	Zinc finger, matrin type 1	ZMAT1	X	Xq21
23	3103	3.751374641	8.308802907	11.89759629	1.45E-05	0.005860743	3.785551986	IMAGE:239287	239287	IMAGE:239287	Hs.192039	Protein tyrosine phosphatase, receptor type, C	PTPRC	1	1q31-q32
24	5210	2.936127849	9.272976056	10.04636305	1.52E-05	0.005860743	3.768513387	IMAGE:324122	324122	IMAGE:324122	Hs.129944	Endothelial cell-specific molecule 1	ESM1	5	5q11.2
25	1805	3.266771833	7.494678638	10.01951779	1.55E-05	0.005860743	3.750948976	IMAGE:45785	45785	IMAGE:45785	Hs.190621	Contactin associated protein-like 2	CNTNAP2	7	7q35-q36
26	2958	2.47096719	9.708742183	9.994892895	1.57E-05	0.005860743	3.734789606	IMAGE:230376	230376	IMAGE:230376	Hs.69771	Complement factor B	CFB	6	6p21.3
27	2770	3.33019536	7.842379244	9.828005009	1.76E-05	0.006336258	3.62405785	IMAGE:213502	213502	IMAGE:213502	Hs.443057	CD53 molecule	CD53	1	1p13
28	630	2.892929914	10.04376366	9.763444936	1.85E-05	0.006392729	3.580645541	IMAGE:124956	124956	IMAGE:124956	Hs.515785	Biliverdin reductase B (flavin reductase (NADPH))	BLVRB	19	19q13.1-q13.2
29	3262	3.273372818	8.164500658	9.683255198	1.95E-05	0.006531444	3.526268758	IMAGE:246564	246564	IMAGE:246564	In multiple clusters				
30	9094	3.581110295	11.64395898	9.557980851	2.14E-05	0.006902645	3.440298148	IMAGE:488870	488870	IMAGE:488870	Hs.1908	Proteoglycan 1, secretory granule	PRG1	10	10q22.1
31	7592	2.380810167	8.926875531	9.46800207	2.28E-05	0.007126326	3.377768866	IMAGE:429349	429349	IMAGE:429349	Hs.386726	Regulator of G-protein signalling 4	RGS4	1	1q23.3
32	7393	2.699282326	8.37023586	9.299531341	2.58E-05	0.007589994	3.258903459	IMAGE:427929	427929	IMAGE:427929	In multiple clusters				
33	7783	3.02438537	7.725141648	9.295429988	2.58E-05	0.007589994	3.255980248	IMAGE:469549	469549	IMAGE:469549	Hs.523443	Hemoglobin, beta	HBB	11	11p15.5
34	2529	3.127650555	9.73673523	8.983937911	3.26E-05	0.009288415	3.02976854	IMAGE:197626	197626	IMAGE:197626	Hs.200230	Family with sequence similarity 102, member B	FAM102B	1	1p13.3
35	1925	4.273413077	8.495704622	8.796774317	3.76E-05	0.010124551	2.889756217	IMAGE:48451	48451	IMAGE:48451	Data not found				
36	9607	2.788475094	8.964153986	8.795785414	3.76E-05	0.010124551	2.889008076	IMAGE:510372	510372	IMAGE:510372	Hs.5940	Mucin 13, cell surface associated	MUC13	3	
37	1555	3.380466854	10.28404427	8.744664661	3.91E-05	0.010246767	2.850212087	IMAGE:151144	151144	IMAGE:151144	Hs.203717	Fibronectin 1	FN1	2	2q34
38	7848	3.523400672	10.87413848	8.596429751	4.39E-05	0.011196994	2.736355598	IMAGE:470007	470007	IMAGE:470007	Hs.76224	EGF-containing fibulin-like extracellular matrix protein 1	EFEMP1	2	2p16
39	1203	2.451971698	10.95903377	8.100967959	6.53E-05	0.015503318	2.340580384	IMAGE:130532	130532	IMAGE:130532	In multiple clusters				
40	1402	1.580250722	9.733877349	8.078706171	6.66E-05	0.015503318	2.322228125	IMAGE:143059	143059	IMAGE:143059	Hs.1048	KIT ligand	KITLG	12	12q22
41	4654	2.912924762	8.224907788	8.03211873	6.92E-05	0.015503318	2.283658953	IMAGE:300203	300203	IMAGE:300203	In multiple clusters				
42	2888	1.618421434	9.274453873	8.021945347	6.98E-05	0.015503318	2.275207025	IMAGE:221773	221773	IMAGE:221773	Hs.50499	Spermatogenesis associated 9	SPATA9	5	5q15
43	9215	2.794817945	7.856274748	8.020922094	6.98E-05	0.015503318	2.274356331	IMAGE:489127	489127	IMAGE:489127	Hs.522891	Chemokine (C-X-C motif) ligand 12 (stromal cell-derived factor 1)	CXCL12	10	10q11.1
44	4128	2.063669977	8.792534596	8.011514734	7.04E-05	0.015503318	2.266530371	IMAGE:286732	286732	IMAGE:286732	Hs.436792	LIM domain only 4	LMO4	1	1p22.3
45	3479	1.414910635	9.017637138	7.927590185	7.55E-05	0.01623988	2.196309806	IMAGE:260052	260052	IMAGE:260052	Hs.14601	Hematopoietic cell-specific Lyn substrate 1	HCLS1	3	3q13
46	2717	1.478547388	11.03690129	7.90277084	7.71E-05	0.01623988	2.175403145	IMAGE:209731	209731	IMAGE:209731	In multiple clusters				
47	1875	3.070011054	8.848676295	8.769336695	9.04E-05	0.018254397	2.132005728	IMAGE:47481	47481	IMAGE:47481	Hs.85201	C-type lectin domain family 2, member B	CLEC2B	12	12p13-p12
48	3839	3.341819763	8.428063863	7.728064062	8.94E-05	0.018254397	2.026402299	IMAGE:278993	278993	IMAGE:278993	Hs.504352	Neurotrimin	HNT	11	11q25
49	1253	3.601563887	10.52799999	7.466257716	0.000112153	0.022187987	1.796950435	IMAGE:135118	135118	IMAGE:135118	Hs.524134	GATA binding protein 3	GATA3	10	10p15
50	1935	3.85462355	8.032555055	7.409408522	0.000117922	0.022723041	1.746123668	IMAGE:49164	49164	IMAGE:49164	Hs.109225	Vascular cell adhesion molecule 1	VCAM1	1	1p32-p31
51	6531	1.966823819	8.83803315	7.393963682	0.000119546	0.022723041	1.732252094	IMAGE:375774	375774	IMAGE:375774	Hs.434973	Glycophorin A (MNS blood group)	GYPA	4	4q28.2-q31.1
52	8832	3.838759032	10.74220659	7.265273845	0.000134096	0.024998638	1.615614987	IMAGE:487878	487878	IMAGE:487878	Hs.111779	Secreted protein, acidic, cysteine-rich (osteonectin)	SPARC	5	5q31.3-q32
53	3478	1.480511617	8.372796485	7.229732223	0.000138457	0.025324614	1.583066645	IMAGE:260200	260200	IMAGE:260200	Hs.153837	Myeloid cell nuclear differentiation antigen	MNDA	1	1q22
54	5143	2.308936834	8.895469608	7.065071334	0.000160847	0.02887501	1.430346368	IMAGE:323181	323181	IMAGE:323181	Hs.516493	Fibroblast activation protein, alpha	FAP	2	2q23
55	5925	1.89707639	7.999096109	7.024031572	0.00016704	0.029263218	1.391783042	IMAGE:358539	358539	IMAGE:358539	Hs.522795	Rac/Cdc42 guanine nucleotide exchange factor (GEF) 6	ARHGEF6	X	Xq26
56	1951	1.636548794	7.977273943	7.011097163	0.000169047	0.029263218	1.379587344	IMAGE:49340	49340	IMAGE:49340	Hs.553147	CDNA FLJ37082 fis, clone BRACE2016465		8	
57	5320	1.408358368	8.824803665	6.98545901	0.000173105	0.029439987	1.355354014	IMAGE:327715	327715	IMAGE:327715	Hs.632594	Glycophorin B (MNS blood group)	GYPB		4q28-q31
58	2758	2.96144723	8.368227257	6.96415628	0.00017656	0.02950984	1.335158276	IMAGE:212423	212423	IMAGE:212423	Hs.90061	Progesterone receptor membrane component 1	PGRMC1	X	Xq22-q24
59	6623	1.50762133	8.857721317	6.931128625	0.00018207	0.029914988	1.303738301	IMAGE:376485	376485	IMAGE:376485	Hs.437322	Tumor necrosis factor, alpha-induced protein 6	TNFAIP6	2	2q23.3
60	8358	2.947130993	10.4035643	6.881457517	0.000190722	0.030814346	1.256235105	IMAGE:485875	485875	IMAGE:485875	Hs.76224	EGF-containing fibulin-like extracellular matrix protein 1	EFEMP1	2	2p16
61	1490	3.621644786	9.201841127	6.8495994	0.000196513	0.031229522	1.225608504	IMAGE:148435	148435	IMAGE:148435	In multiple clusters				
62	7638	3.961360935	10.16533014	6.821187633	0.000201844	0.031559344	1.198189663	IMAGE:429771	429771	IMAGE:429771	Hs.76392	Aldehyde dehydrogenase 1 family, member A1	ALDH1A1	9	9q21.13
63	6548	1.826293325	8.525372445	6.700903	0.000226277	0.033665042	1.080998115	IMAGE:376134	376134	IMAGE:376134	Hs.76152	Aquaporin 1 (Colton blood group)	AQP1	7	7p14
64	9217	1.999155689	6.982582423	6.677332867	0.000231443	0.033665042	1.057821731	IMAGE:489099	489099	IMAGE:489099	Hs.489142	Collagen, type I, alpha 2	COL1A2	7	7q22.1
65	2764	1.233556096	8.540637721	6.675492491	0.000231851	0.033665042	1.056009149	IMAGE:212688	212688	IMAGE:212688	In multiple clusters				
**66**	**3580**	**2.416989116**	**8.438698359**	**6.675084953**	**0.000231942**	**0.033665042**	**1.055607708**	**IMAGE:268652**	**268652**	**IMAGE:268652**	**Hs.370771**	**Cyclin-dependent kinase inhibitor 1A (p21, Cip1)**	**CDKN1A**	**6**	**6p21.2**

67	7441	1.228293353	9.658878128	6.667863754	0.000233555	0.033665042	1.04849106	IMAGE:428404	428404	IMAGE:428404	Hs.81170	Pim-1 oncogene	PIM1	6	6p21.2
68	5905	1.303951426	8.847929786	6.647444206	0.000238183	0.033665042	1.028331439	IMAGE:358506	358506	IMAGE:358506	Hs.368921	Collagen, type XVI, alpha 1	COL16A1	1	1p35-p34
69	1809	2.476280484	7.840633311	6.63678172	0.000240641	0.033665042	1.017783669	IMAGE:46408	46408	IMAGE:46408	Hs.559718	Adenylate kinase 5	AK5	1	1p31
70	4390	1.362927378	8.331019129	6.626133977	0.000243123	0.033665042	1.007236068	IMAGE:292699	292699	IMAGE:292699	Hs.491558	Ankyrin 1, erythrocytic	ANK1	8	8p11.1
71	2694	1.204921315	10.02164736	6.60777945	0.000247469	0.033665042	0.989020281	IMAGE:208012	208012	IMAGE:208012	Data not found				
72	573	2.474226244	8.553088487	6.597095304	0.00025004	0.033665042	0.97839711	IMAGE:121114	121114	IMAGE:121114	Data not found				
73	1760	2.328495851	10.06288791	6.510545695	0.000271993	0.036119161	0.891801692	IMAGE:44449	44449	IMAGE:44449	Hs.504352	Neurotrimin	HNT	11	11q25
74	5353	2.990560937	11.3139026	6.39206676	0.000305616	0.040035731	0.771685713	IMAGE:328692	328692	IMAGE:328692	Hs.624	Interleukin 8	IL8	4	4q13-q21
75	7872	1.794841872	7.666567144	6.365134707	0.000313891	0.040319622	0.744124894	IMAGE:470057	470057	IMAGE:470057	Hs.23871	CDNA clone IMAGE:30924414		5	
76	3303	1.341397077	7.112858311	6.358072785	0.000316102	0.040319622	0.736882255	IMAGE:248463	248463	IMAGE:248463	Hs.585357	Hemoglobin, zeta	HBZ	16	16p13.3
77	485	2.149281831	7.192430667	6.336364222	0.000323008	0.040665403	0.714576829	IMAGE:115277	115277	IMAGE:115277	Hs.513440	G protein-coupled receptor 65	GPR65	14	14q31-q32.1
78	3304	1.933295895	11.05203611	6.279293052	0.000341979	0.04238959	0.655637773	IMAGE:248589	248589	IMAGE:248589	Hs.510078	Serum/glucocorticoid regulated kinase	SGK	6	6q23
79	1428	1.32947975	10.28647916	6.269238826	0.000345448	0.04238959	0.645209451	IMAGE:144805	144805	IMAGE:144805	Hs.417022	Intestinal cell (MAK-like) kinase	ICK	6	6p12.3-p11.2
80	2000	2.505757302	9.781003776	6.22789593	0.000360132	0.04363904	0.602185781	IMAGE:50243	50243	IMAGE:50243	Hs.411391	Hypothetical gene supported by BX647608	LOC399959	11	11q24.1
81	3520	1.801720147	9.33778349	6.205511848	0.000368372	0.043908414	0.578795705	IMAGE:263845	263845	IMAGE:263845	Hs.495710	Glycoprotein M6B	GPM6B	X	Xp22.2
82	7582	3.221775748	9.341449474	6.194968836	0.000372325	0.043908414	0.567755428	IMAGE:429203	429203	IMAGE:429203	In multiple clusters				
83	3672	-1.379340109	11.52285624	-6.185428731	0.000375944	0.043908414	0.557752399	IMAGE:272525	272525	IMAGE:272525	Hs.442782	Chromosome 14 open reading frame 94	C14orf94	14	14q11.2
84	6771	1.709447965	8.502255678	6.05702162	0.000428656	0.049468978	0.421908207	IMAGE:377446	377446	IMAGE:377446	Hs.2257	Vitronectin	VTN	17	17q11
85	5900	1.310887227	11.25724317	6.036140806	0.000437983	0.049788539	0.399604231	IMAGE:358168	358168	IMAGE:358168	Hs.507584	Hypothetical protein MGC9850	MGC9850	13	13q12.2
86	4041	1.654804089	8.42831301	6.027961001	0.000441697	0.049788539	0.390850504	IMAGE:284459	284459	IMAGE:284459	Hs.460355	Protein kinase C, beta 1	PRKCB1	16	16p11.2
87	9691	3.520661169	12.65904677	5.99198198	0.000458459	0.049914437	0.352237049	IMAGE:512287	512287	IMAGE:512287	In multiple clusters				
88	6849	1.346301267	11.67474468	5.972111789	0.000468018	0.049914437	0.33083487	IMAGE:380600	380600	IMAGE:380600	Hs.435735	Solute carrier family 5 (sodium-dependent vitamin transporter), member 6	SLC5A6	2	2p23
89	8139	1.106269101	9.022424595	5.969495786	0.000469293	0.049914437	0.328013079	IMAGE:471725	471725	IMAGE:471725	Hs.567295	Inositol 1,4,5-triphosphate receptor, type 1	ITPR1	3	3p26-p25
90	7880	2.353592367	9.536587449	5.952621478	0.000477611	0.049914437	0.309788414	IMAGE:469999	469999	IMAGE:469999	Hs.567498	Chromosome 4 open reading frame 18	C4orf18	4	4q32.1
91	7158	1.318197133	9.184291278	5.949716171	0.00047906	0.049914437	0.3066466	IMAGE:416854	416854	IMAGE:416854	Hs.156540	Secretogranin V (7B2 protein)	SCG5	15	15q13-q14
92	4135	1.973624953	8.202460631	5.938647576	0.000484623	0.049914437	0.294666155	IMAGE:286899	286899	IMAGE:286899	Hs.500695	Ligand dependent nuclear receptor corepressor	LCOR	10	10q24
93	1005	1.22708184	11.29332784	5.932558281	0.000487715	0.049914437	0.288067905	IMAGE:36809	36809	IMAGE:36809	Hs.148909	Cell adhesion molecule with homology to L1CAM (close homolog of L1)	CHL1	3	3p26.1
94	9106	1.311475125	11.29175314	5.931815719	0.000488093	0.049914437	0.287262923	IMAGE:489048	489048	IMAGE:489048	Data not found				
95	6556	3.167242192	12.2815269	5.922688349	0.000492772	0.049914437	0.277361993	IMAGE:376178	376178	IMAGE:376178	Hs.530862	Protein kinase, AMP-activated, gamma 1 non-catalytic subunit	PRKAG1	12	12q12-q14
96	622	2.459890647	9.331898196	5.919720839	0.000494304	0.049914437	0.274140467	IMAGE:124543	124543	IMAGE:124543	In multiple clusters				
**97**	**5994**	**3.562090457**	**12.27130942**	**5.900384292**	**0.000504418**	**0.050053398**	**0.253118455**	**IMAGE:359412**	**359412**	**IMAGE:359412**	**Hs.376071**	**Cyclin D2**	**CCND2**	**12**	**12p13**
98	8191	1.45466003	9.987670227	5.8973843	0.000506007	0.050053398	0.249852262	IMAGE:472138	472138	IMAGE:472138	Hs.510078	Serum/glucocorticoid regulated kinase	SGK	6	6q23
99	5646	-1.039254176	9.865939186	-5.848204273	0.000532876	0.051687545	0.196127472	IMAGE:346445	346445	IMAGE:346445	Hs.244391	Zinc finger protein 582	ZNF582	19	19q13.43
100	5870	2.725224028	13.05284184	5.847643441	0.000533191	0.051687545	0.195512843	IMAGE:357775	357775	IMAGE:357775	Hs.438863	Nuclear receptor subfamily 1, group H, member 3	NR1H3	11	11p11.2
