# Supplementary material for: p21 promotes oncolytic adenoviral activity in ovarian cancer and is a potential biomarker
Source: Mol Cancer. 2010 Jul 3;9:175. doi: 10.1186/1476-4598-9-175 (PMC2904726; doi:10.1186/1476-4598-9-175)
Supplement: Additional file 5 — Supplementary figure 4. Replication of dl922-947 in HCT116 p21+/+ and p21-/- cells. [file 1476-4598-9-175-S5.PDF]

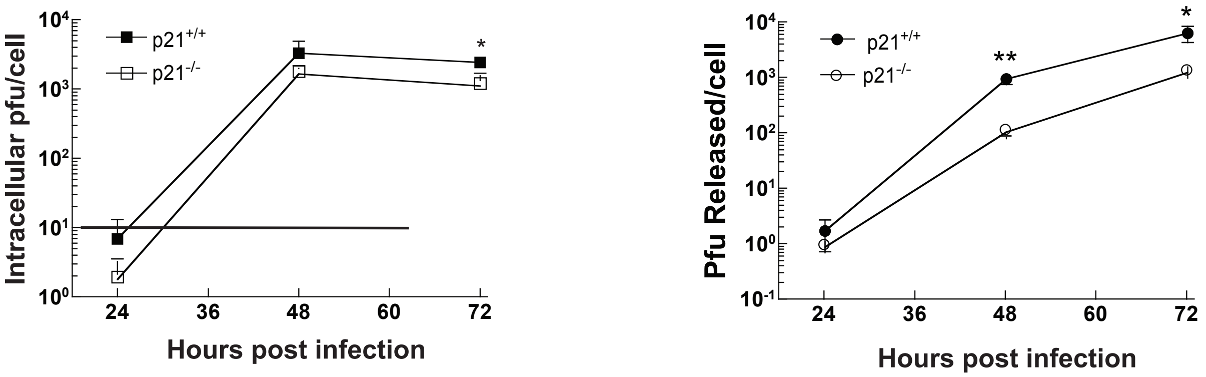

Supplementary Figure 4: Hct116 p21+/+ and p21-/- cells were infected with *dl*922-947 (MOI 10). Intracellular viral replication (left) and titre of virus released into supernatant (right) was assessed up to 72h pi by TCID50 assay. Horizontal line represents input dose. *; $p < 0.05$. **; $p < 0.01$.
