# Supplementary material for: p21 promotes oncolytic adenoviral activity in ovarian cancer and is a potential biomarker
Source: Mol Cancer. 2010 Jul 3;9:175. doi: 10.1186/1476-4598-9-175 (PMC2904726; doi:10.1186/1476-4598-9-175)
Supplement: Additional file 6 — Supplementary figure 5. BRCU incorporation in A2780CP and A2780P-p21 cells. [file 1476-4598-9-175-S6.PDF]

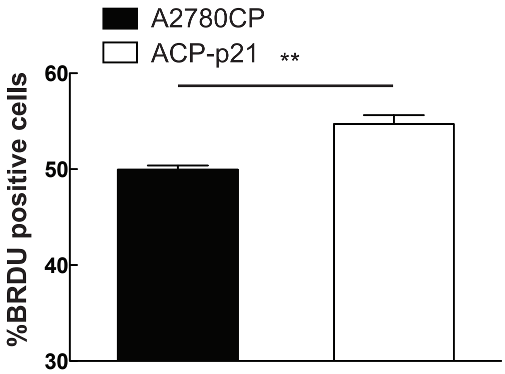

Supplementary Figure 5: DNA replication was assessed in asynchronous parental A2780CP and ACP-p21 cells by BRDU incorporation. ** p = 0.0012
