# Supplementary material for: p21 promotes oncolytic adenoviral activity in ovarian cancer and is a potential biomarker
Source: Mol Cancer. 2010 Jul 3;9:175. doi: 10.1186/1476-4598-9-175 (PMC2904726; doi:10.1186/1476-4598-9-175)
Supplement: Additional file 7 — Supplementary figure 6. Infectivity of A2780CP and A2780CP-p21 cells. [file 1476-4598-9-175-S7.PDF]

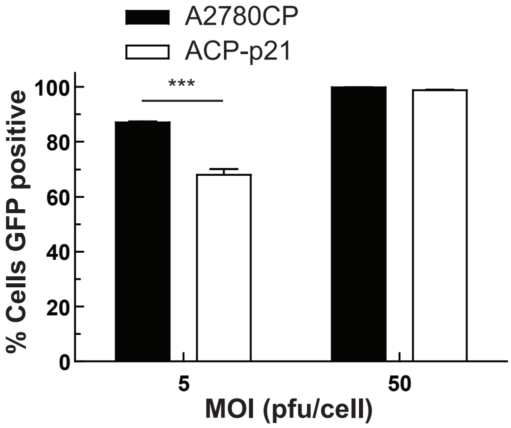

Supplementary Figure 6: Infectivity of parental A2780CP and ACP-p21 cells was assessed by flow cytometry 24h following infection with Ad CMF-GFP (MOI 5 and 50). *** $p < 0.0001$
