# Supplementary material for: p21 promotes oncolytic adenoviral activity in ovarian cancer and is a potential biomarker
Source: Mol Cancer. 2010 Jul 3;9:175. doi: 10.1186/1476-4598-9-175 (PMC2904726; doi:10.1186/1476-4598-9-175)
Supplement: Additional file 8 — Supplementary figure 7. Basal AKT phosphorylation in four ovarian cancer cell lines. [file 1476-4598-9-175-S8.PDF]

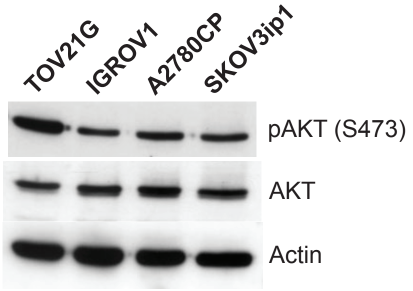

Supplementary Figure 7: TOV21G, IGROV1, A2780CP and SKOV3ip1 cells were plated overnight in serum-free medium prior to harvesting. Expression of total AKT and phospho-AKT (Ser 473) was assessed by immunoblot.
