# Supplementary material for: p21 promotes oncolytic adenoviral activity in ovarian cancer and is a potential biomarker
Source: Mol Cancer. 2010 Jul 3;9:175. doi: 10.1186/1476-4598-9-175 (PMC2904726; doi:10.1186/1476-4598-9-175)
Supplement: Additional file 9 — Supplementary figure 8. Basal Histone H1 phosphorylation in four ovarian cancer cell lines [file 1476-4598-9-175-S9.PDF]

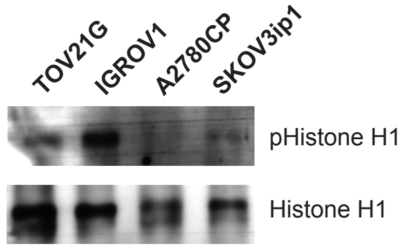

Supplementary Figure 8: Following acid extraction, expression of phospho-Histone H1 and total Histone H1 was assessed in asynchronous, logarithmically growing TOV21G, IGROV1, A2780CP and SKOV3ip1 cells by immunoblot.
