# Supplementary material for: p21 promotes oncolytic adenoviral activity in ovarian cancer and is a potential biomarker
Source: Mol Cancer. 2010 Jul 3;9:175. doi: 10.1186/1476-4598-9-175 (PMC2904726; doi:10.1186/1476-4598-9-175)
Supplement: Additional file 10 — Supplementary materials and methods. Method for acid histone extraction as well as techniques used for cell line verification. [file 1476-4598-9-175-S10.DOC]

**Supplementary Materials and Methods**

All cell lines were tested for mycoplasma every two weeks. The source and date of acquisition of cells was as follows: A2780CP, Dr A. Eliopoulos (University of Birmingham, UK), 1998; IGROV1, Dr M. Ford (Glaxo-Wellcome, UK), 1999; OVCAR4 and OVCAR5, Dr R. Camalier (NCI, Frederick, MD), 1999; OVCAR3 and SKOV3, Cancer Research UK Cell Services, 2001; MRC5 and MRC5-VA, Cancer Research UK Cell Services, 2008; Hct116 p21^+/+^ and p21^-/-^ cells, the lab of Dr Bert Vogelstein, 2007; TOV21G, Dr S. Gayther (UCL, London), 2004; SKOV3ip1 cells were a gift from their creator, Dr J Price, University of Texas MD Anderson Cancer Center in 1999. IOSE25 cells were created in the lab of one of us (FRB) in 2006 and their characteristics and those of TOSE1 and TOSE 4 cells are described elsewhere (Archibald et al MS in preparation). Cells were authenticated as follows: the resistance to cisplatin of A2780CP cells compared to parental A2780 cells was tested every 6 months; SKOV3, SKOV3ip1, IGROV1 and TOV21G cells underwent SNP array and short tandem repeat analyses in 2008; Hct116 p21^+/+^ and p21^-/-^ cells were analysed monthly for p21 expression by immunoblotting.

Histones were extracted as follows. Cells were lysed on ice for 10 minutes in Triton extraction buffer (0.5% Triton X-100 in PBS supplemented with protease inhibitors (Roche) and phosphatase inhibitors (PhosStop, Roche). After centrifugation, histones were extracted overnight at 4 ^o^C in 100µl 0.2N HCl. Expression of phospho-histone H1 and Histone H1 was assessed by standard immunoblot.
